# Improving plant-based genotoxicity bioassay through AFLP technique for trace metal-contaminated water: insights from *Myriophyllum aquaticum* (Vell.) Verdc. and Cd

**DOI:** 10.1007/s11356-022-19429-y

**Published:** 2022-03-10

**Authors:** Andrea Coppi, Ilaria Colzi, Lorenzo Lastrucci, Maria Beatrice Castellani, Cristina Gonnelli

**Affiliations:** 1 Department of Biology, Università Degli Studi Di Firenze, via Micheli 1, Florence, 50121 Italy; 2 University Museum System, Natural History Museum, Botany, via La Pira 4, Florence, 50121 Italy

**Keywords:** AFLP, Aquatic plants, Bioassays, Genotoxicity, Trace metals

## Abstract

In this work, we evaluated whether the species *Myriophyllum aquaticum* (Vell.) Verdc. can be a promising material for devising reliable eco-toxicological tests for Cd-contaminated waters. Plants of *M. aquaticum* were exposed to Cd, using different concentrations (1 mg L^−1^, 2.5 mg L^−1^, 5 mg L^−1^, and 10 mg L^−1^; experiment 1) and exposure times (2.5 mg L^−1^ for 3 days, 7 days, 14 days, and 21 days; experiment 2). Plant growth and Cd accumulation were monitored during the treatment period, and Cd genotoxicity was assessed by analyzing Cd-induced changes in the AFLP fingerprinting profiles using famEcoRI_(TAC)_/MseI_(ATG)_ and hexEcoRI_(ACG)_/MseI_(ATG)_ pairs of primers. Root and shoot growth was reduced already at the lowest Cd concentration used (about 20% reduction for roots and 60% for shoots at 1 mg L^−1^; experiment 1) and after 7 days (about 50% reduction for roots and 70% for shoots; experiment 2). The primer combinations produced 154 and 191 polymorphic loci for experiments 1 and 2, respectively. Mean genetic diversity (He) reduction among the treatment groups was observed starting from 2.5 mg L^−1^ (He 0.211 treated vs 0.236 control; experiment 1) and after 3 days (He 0.169 treated vs 0.261 control; experiment 2), indicating that results obtained from AFLP profiles did not match with plant growth measurements. Therefore, our results showed that *M. aquaticum* proved to be a suitable model system for the investigation of Cd genotoxicity through AFLP fingerprinting profile, whereas the more classic eco-toxicological tests based only on biometric parameters could not correctly estimate the risk associated with undetected Cd genotoxicity.

## Introduction

Over the past decades, intensive industrialization and agriculture have increased considerably the release of various toxic compounds into air, soil, and water, causing many environmental problems (Khan and Ghouri 2011). Among the most common contaminants, trace metals are of serious concern since, unlike organic toxins, they are non-biodegradable and accumulate in the environmental matrixes (Ali et al. 2013). Water, the key vital resource for natural ecosystems and human life, is subjected to a continuous anthropogenic input of these elements that can thus freely reach any kind of biota (Schwarzenbach et al. 2006). Therefore, the development of scientifically sound and cost-efficient tools for contaminant monitoring is a priority for the overall protection of all living organisms, along with the pressing necessity to restore the polluted aquatic ecosystems and to clean the contaminated wastewaters.

Among the monitoring tools, effect-based methods, such as bioassays and biomarkers, are widely employed because of their unique ability to establish the relationship between chemical pollution and ecological status, covering a broad range of exposure times and toxicity mechanisms in diverse biological systems (Brack et al. 2017). Test organisms include invertebrates, fishes, microorganisms, plants, and algae, even though some of them can be difficult to handle and their use may be ethically objectionable. Other systems, such as mammalian cells, are expensive, and results are not always consistent (Hassan et al. 2016). Nonetheless, the use of aquatic plants as biological models in eco-toxicological tests is limited if compared to animals, although contaminants mainly enter the ecosystem through such organisms that are the first and obligate step of the trophic chains (Ceschin et al. 2020).

In recent years, increasing attention has been paid to bioassays for the assessment of trace metal genotoxic effects since the interaction with nucleic acids is considered one of the primary causes of the toxicity of such elements (Kleinjans and van Schooten 2002; Zhu and Costa 2020). In practice, only when genotoxicity assays are added to the analysis of conventional water quality parameters, the presence of mutagens in water is considered to be reliably assessed (Ohe et al. 2004). As for the test organisms, plants are regarded as ideal assay systems for screening and monitoring mutagens in the environment, providing important information in efforts to conserve biodiversity and ecological resources (Panda and Panda 2002; Aksoy 2017). Plants are affected by water pollution earlier than other organisms since they are the first interface between abiotic and biotic constituents of an ecosystem and, therefore, considered as early warning systems, essential for intercepting contaminations in advance (Ceschin et al. 2020). The development of molecular biology has led to several PCR-based techniques used to evaluate DNA damage in toxicological studies with plants as model systems. The techniques include analysis of microsatellite markers (Monteiro et al. 2009), random amplified polymorphic DNA assay (Liu et al. 2009; Surgun-Acar et al. 2018), and analysis of amplified fragment length polymorphism (AFLP). In plant research, the AFLP technique is already a powerful tool with broad applications in population genetics, linkage mapping, phylogeny, and biogeography (Meudt and Clarke 2007). More recently, AFLP has been used to screen plant genomic DNA for evidence of mutational events induced by environmental contaminants (Labra et al. 2003; Aina et al. 2007; Coppi et al. 2018; Tanee et al. 2018).

Among the most dangerous mutagens, cadmium (Cd) is one of the trace elements of concern for the environment and human health (ATSDR 2007), thus needing to be extensively studied and monitored for its public health effects (ATSDR 2015; USEPA 2015). The genotoxicity of Cd, classified as human carcinogen (IARC 2016), is supposed to derive from its direct binding to DNA, possibly at adenine, guanine, and thymine (Hossain and Huq 2002), or direct inhibition of DNA mismatch repair (Jin et al. 2003). The Cd genotoxic effect may also be indirect, through the generation of reactive oxygen species, which may then damage nucleic acids (Apel and Hirt 2004; Valverde et al. 2001). Several studies have demonstrated Cd-induced micronuclei formation, chromosomal aberrations, or DNA base damage (Beyersmann and Hartwig 2008). Therefore, the assessment of genotoxicity of metals such as Cd is an important topic in environmental research, with the increasing need to design eco-toxicological tests sensitive to both concentration and exposure time to capture and address any possible effect of its presence. To this aim, we performed a toxicity test to assess if the species *Myriophyllum aquaticum* (Vell.) Verdc. can be a promising model system to propose to the eco-toxicologist scientific community in the view of devising reliable bioassays for Cd genotoxicity in waters. *Myriophyllum aquaticum* is a macrophyte native to tropical and subtropical America. We chose this species because of its unique advantages of year-round availability, wide distribution, easy to handle, and to grow without the need for sterile conditions or expensive materials. Furthermore, an aquatic species can represent per se a more suitable model system for the evaluation of the genotoxic potentiality of contaminated waters, whereas generally, such kinds of bioassays are generally performed on land plants. In addition, this plant can be easily propagated in a vegetative way, thus providing abundant live test material with a low level of genetic variability that should be more reliable in revealing variation in its genetic structure when exposed to Cd. Considering *M. aquaticum* as a promising model system, we assessed the changes caused by toxic Cd concentration on its AFLP fingerprinting profiles at different doses and times of exposure. We moreover tested if one of the response traits of plants to the exposure of toxic concentration of Cd (e.g. plant growth) is time-coupled to evident genotoxic effect. Therefore, our results could provide fundamental information to design not only more reliable eco-toxicological tests but also on the still poorly known mechanism of Cd genotoxic effects on plants.

## Materials and methods

### Plant material sampling and acclimatization conditions

Stems of *M. aquaticum* were collected from Lago di Porta (PO) (Lastrucci et al. 2017) and cultivated in tap water, as described in Colzi et al. (2018). After one week of acclimation in a greenhouse, plants were propagated by cutting apical segments of uniform size (about 5 cm, three internodes) and cultivated in deionized water in 10-L tanks in a growth chamber (24/16 ℃ day/night; light intensity 100 μmol m^−2^ s^−1^, 12 h d^−1^; relative humidity 60–65%). After two weeks of acclimatization, homogeneous plants (about 2 g f.w.) were selected for the following experimental procedures.

### Experiment 1: Cd treatment at different metal concentrations

60 plants were transferred in 1-L polyethylene pots (one plant per pot, 12 pots per treatment) containing control solutions alone or added with 1 mg L^−1^, 2.5 mg L^−1^, 5 mg L^−1^, and 10 mg L^−1^ of Cd, administered as CdSO_4_. Media and procedures for the experiments were adapted from OECD protocols for *M. spicatum* (OECD 2014). Exposure duration was shortened to seven days to intercept in advance possible genotoxic effects. The treatment levels were selected to have, in the plant growth solutions, Cd concentrations exceeding the guidance levels for water quality standard in surface freshwater, established by national legislation (D.lgs. 152/2006). Furthermore, we used Cd concentrations in the same range of Wang et al. (2021), who showed them as environmentally significant, in addition to two higher concentrations (5 mg L^−1^ and 10 mg L^−1^), since, reviewing the literature, Cd concentrations in wastewaters was found as high as 20 mg L^−1^ (Salameh et al., 2018). The pots were maintained in the growth chamber throughout the duration of the treatment, and after seven days of exposure, the plants were sampled for growth evaluation, determination of metal concentration, and molecular analysis.

### Experiment 2: Cd treatment at different exposure times

96 plants were transferred in 1-L polyethylene pots (one plant per pot, 12 pots per treatment) containing control solutions alone or with 2.5 mg L^−1^ of Cd administered as CdSO_4_. The Cd dose used for the treatment was established from the results of the previous experiment that used different concentrations.

The pots were maintained in the growth chamber throughout the duration of the treatment, removing plants from the test solution after 3 days, 7 days, 14 days, and 21 days of exposure, respectively, for evaluation.

### Growth measurement and determination of Cd concentration

Root and shoot length and fresh weight of each plant were measured prior to and after Cd treatment to evaluate plant response to Cd concentration and exposure time in terms of increment in plant growth. Subsequently, each sample was incubated in ice-cold (4 ℃) Pb(NO_3_)_2_ (10 mM) for 30 min, as in Barzanti et al. (2011), to remove the adsorbed metal from the cell wall, and rinsed with milliQ-water. Roots and shoots were then separated and oven-dried for 24 h at 80 ℃. For the Cd concentration analysis, aliquots of oven-dried material were weighed (about 100 mg) and mineralized with concentrated HNO_3_ (65%) at 200 ℃ for 20 min in a microwave digestion system (Mars 6, CEM, Matthews, North Carolina, USA) as in Bettarini et al. (2019). After digestion, the solutions were adjusted to a volume of 25 mL with deionized water and Cd concentrations were determined by atomic absorption spectrophotometry (AAnalyst 200, Perkin Elmer). Certified reference materials (LGC No 7162) were used to verify the accuracy and precision of the methods.

### DNA isolation and AFLP protocol

A portion of shoot material (apex) was sampled from each plant, after growth measurements, from both experiments 1 and 2; 30 mg of each dried plant portion were ground in a mortar with sterile sand. The DNA was extracted by using the 2xCTAB protocol (Doyle and Doyle 1990). The quality and quantity of the extracted DNA were checked by a spectrometric survey that used a Bio-Photometer (Eppendorf). The AFLP analysis followed standard procedure with minor modifications (Coppi et al. 2018). The separation of amplification products was performed by capillary electrophoresis (Applied Biosystems 3130xl Genetic Analyzer) using fluorescent-end-labeled primers. In order to screen the primers combination that produces the most informative, readable, and repeatable profiles, three primer pair combinations (Table 1) were tested on at least three individuals from each treatment group.

**Table 1:** List of primer tested for the AFLP analysis

Fluorescent end-labeling	Primer name	Primer sequence
	PMseI_ATG	GATGAGTCCTGAGTAA(ATG)
	PMseI_TTC	GATGAGTCCTGAGTAA(TTC)
5’hexachloro-fluorescein-phosphoramidite	hex_pEcoRI_ACG	GACTGCGTACCAATTC(ACG)
5′ 6-fluorescein amidite	fam_pEcoRI_TAC	GACTGCGTACCAATTC(TAC)
5′ 6-fluorescein amidite	fam_pEcoRI_CAT	GACTGCGTACCAATTC(CAT)

Fluorescent labeling and sequence with selective extension (in brackets) were also added

Two pairs of primers were selected for the final analysis: famEcoRI_(TAC)_/MseI_(ATG)_ and hexEcoRI_(ACG)_/MseI_(ATG)_.

### Analysis of genetic variation at intra- and inter-treatment group level

Within-treatment group, average genetic diversity was expressed as the computed probability that two randomly chosen homologous sites are different (Nei 1987) by using the program Arlequin v2.000 (Schneider et al. 2000). Genomic template stability was evaluated by comparing the differences in the percentage of polymorphic loci of each sample’s AFLP profiles within each treatment group. The percentage composition of polymorphic bands was calculated as [(nfrag/ntotal)*100], where nfrag was AFLP loci detected for each sample and ntotal the number of total detected bands for the primer pair.

The identification of specific loci (SpL) was also performed. The SpL were identified as those loci that are not shared but either, present only in the control group or those loci present only in the treated groups. Analysis of molecular variance (AMOVA, Excoffier et al. 1992), implemented in Arlequin v2.000 (Schneider et al., 2000), was used to analyze the partitioning of total genetic variation within each treatment group and among treatment groups. The analyses were performed separately for experiments 1 and 2. Statistical support for the different hypothetical groupings of treatment group, based on treatment, was tested in terms of the variance components and the percentage of total expressed variation. Genetic distances between sampling groups were estimated by computing a Slatkin’s linearized pairwise Fst matrix (Slatkin 1995), which was then used to generate a neighbor-joining (Saitou and Nei 1987) dendrogram with the software Mega 6 (Tamura et al. 2013).

### Statistics

Statistical analysis was carried out with one-way ANOVA using the statistical program SPSS 13.0 (SPSS Inc. Chicago, IL, USA). A posteriori comparison of individual means was performed using Tukey’s test.

Custom-made worksheets and program files for SigmaPlot 10.0 (SPSS, Chicago, IL) were used for the analysis of toxicity data. The half-maximal effective concentration (EC_50_) was calculated on both the Cd concentration present in the medium and in the plant tissues. Reports about plant response to metals only rarely estimate sensitivity using quantitative parameters based on the internal concentration (Galardi et al. 2007; Colzi et al. 2011, 2014), whereas such calculation is necessary for studying the relationship between metal toxicity and accumulation.

## Results

### Plant growth and Cd accumulation

**Fig. 1:**
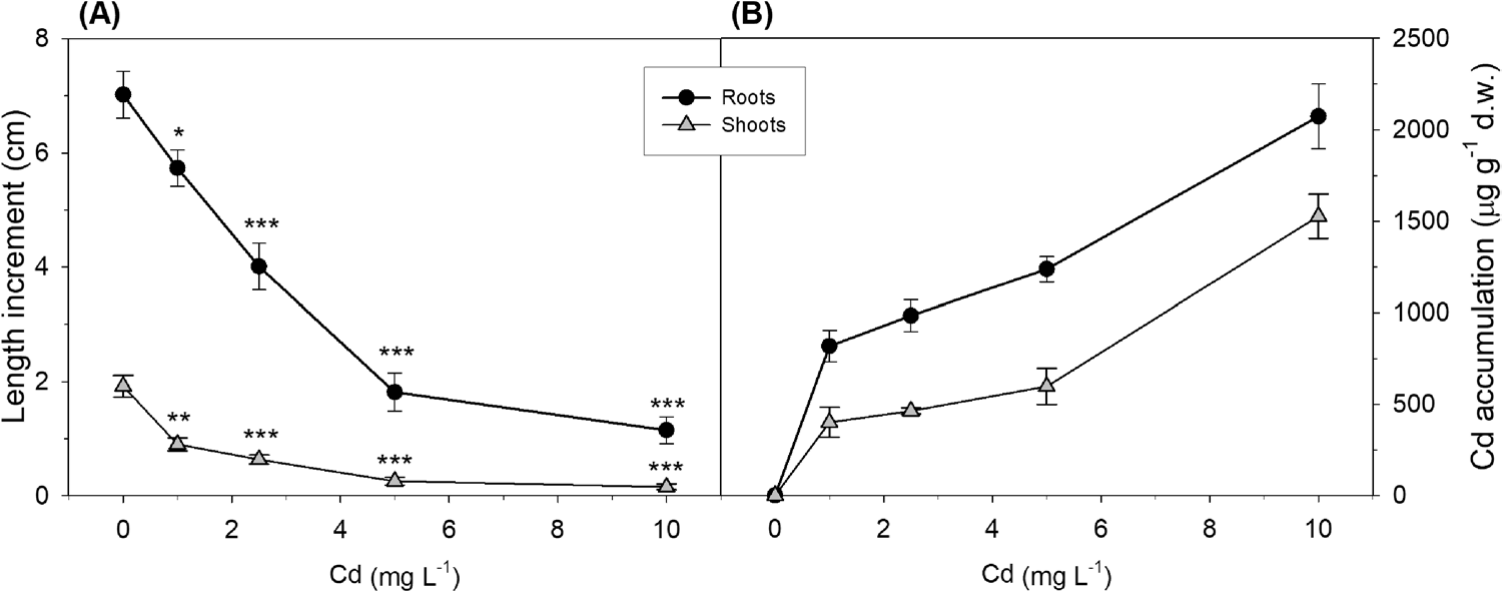
Results of experiment 1: (**A**) root and shoot increment in length (cm) and (**B**) Cd accumulation in roots and shoots (µg g^−1^ d.w.) of *M. aquaticum* after exposure to increasing Cd concentrations for 7 days. Values are mean ± standard error. The significant effect of metal concentration in respect to control is indicated by asterisks (*p < 0.05; **p < 0.01; ***p < 0.001)

Data on plant growth and Cd accumulation in roots and shoots of *M. aquaticum* after Cd exposure are reported in Fig. 1A, B. The presence of the metal induced a significant reduction in both root and shoot length increment starting from the lowest concentration used. A significant fitting of root and shoot growth data to the logistic dose–response curve was obtained (*P* < 0.001, *R* = 0.96 for roots; *P* < 0.001, *R* = 0.91 for shoots). For a quantitative estimation of the Cd effect on plant growth, the parameter EC_50ext_ was calculated on external Cd concentration. The obtained values were 2.5 ± 0.5 mg L^−1^ for roots and 2.0 ± 0.5 mg L^−1^ for shoots and were not significantly different from each other.

Cadmium concentrations in both roots and shoots of *M. aquaticum* increased with increasing metal exposure (Fig. 1B), reaching values of about 1600 µg g^−1^ d.w. and 1200 µg g^−1^ d.w., respectively, at the highest level of exposure used. Cadmium accumulation in the roots was always significantly higher than in the shoots (*p* < 0.05).

**Fig. 2:**
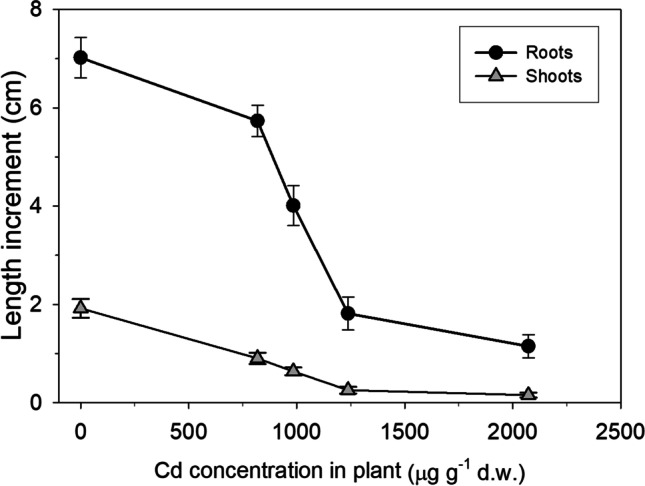
Length increment of roots and shoos (cm) in relation to the internal Cd concentrations (µg g^−1^) accumulated at the end of the experiment in roots and shoots, respectively

The parameter EC_50_ was also calculated on the basis of internal Cd concentration (EC_50int_), using root and shoot accumulation data (Fig. 2). As for external Cd concentration, the data fitting gave significant results for a logistic dose–response relationship (*P* < 0.001, *R* = 0.97 for roots; *P* < 0.0001, *R* = 0.91 for shoots) and the value of EC_50int_ was significantly higher for roots (974 ± 34 µg g^−1^ d.w.) than for shoots (785 ± 71 µg g^−1^ d.w.).

**Fig. 3:**
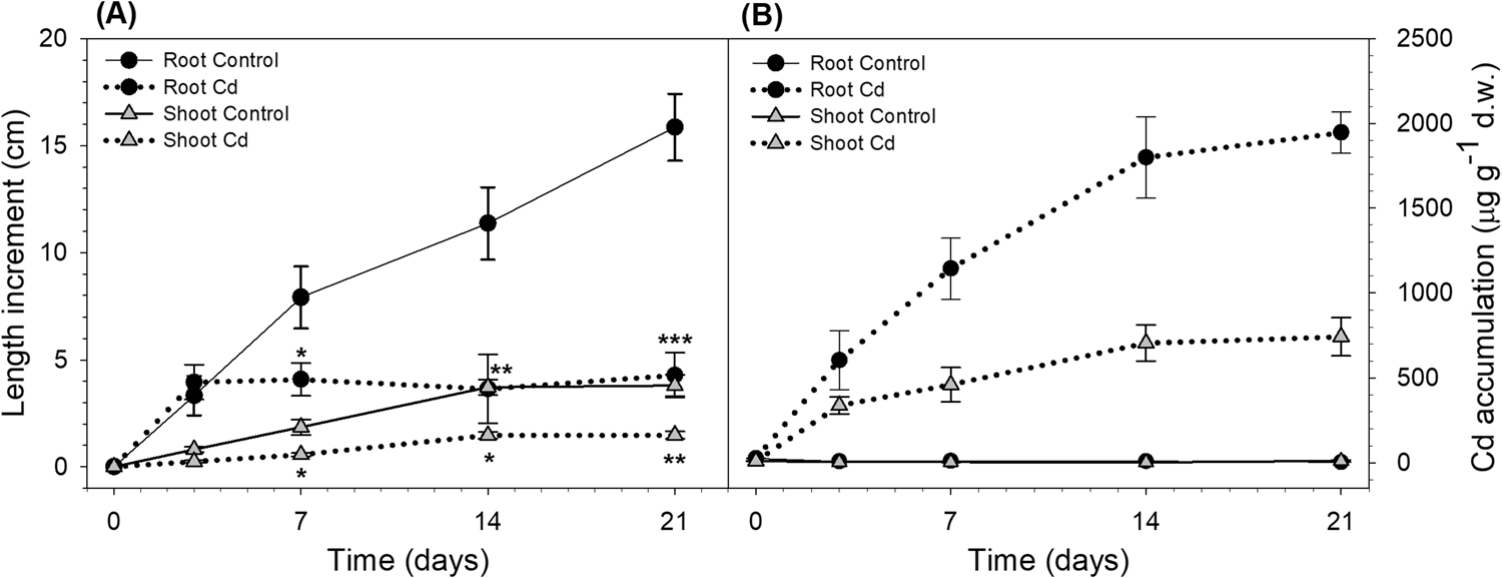
Results of experiment 2: (**A**) root and shoot increment in length (cm) and (**B**) Cd accumulation in roots and shoots (µg g^−1^ d.w.) of *M. aquaticum* after different exposure times (0–21 days) at 2.5 mg L^−1^ Cd. Values are mean ± standard error. The significant effect of Cd treatment in respect to the respective control is indicated by asterisks (*p < 0.05; **p < 0.01; ***p < 0.001)

In experiment 2, plant growth and Cd accumulation at a fixed Cd concentration (2.5 mg L^−1^) were monitored over different exposure times until 21 days (Fig. 3A, B). The presence of Cd in the solution started to produce a significant reduction in root and shoot growth after seven days of exposure (Fig. 3A).

Regarding Cd accumulation, both roots and shoots showed an increase in metal concentration with time. Starting from seven days of exposure, Cd concentrations were always significantly higher in roots than in shoots (at least *p* < 0.01), reaching values about 2.5-fold higher after 14 days and 21 days.

### Analysis of AFLP profiles

AFLP analysis was successfully performed on a total of 124 plants, 52 from experiment 1 and 72 from experiment 2 (Table 2), each of which produced a peculiar fingerprint profile. As for experiment 1, the primer combinations produced a total of 154 polymorphic loci, 84 from the famEcoRI_(TAC)_/MseI_(ATG)_ and 70 from the hexEcoRI_(ACG)_/MseI_(ATG)_. A total of 191 loci were obtained for experiment 2, 88 from the famEcoRI_(TAC)_/MseI_(ATG)_ and 103 from the hexEcoRI_(ACG)_/MseI_(ATG)_. The mean level of genetic diversity within-treatment groups was higher in the control samples (0.236 and 0.261 in experiments 1 and 2, respectively) than the treated samples (0.211 and 0.169 in experiments 1 and 2, respectively). Concerning experiment 1, the levels of genetic diversity within each sampling group varied from 0.256 to 0.161 for TREAT1 and TREAT10, respectively (Table 2).

**Table 2:** Number of analyzed individuals (*n*. sample) for each treatment group (ID) for both experiments

ID	n. sample	Cd (mg L^−1^)	Time (days)	He
***Experiment 1***				
CONT	12	0	7	0.236
TREAT1	10	1	7	0.256
TREAT2.5	12	2.5	7	0.237
TREAT5	11	5	7	0.191
TREAT10	7	10	7	0.161
***Experiment 2***				
CONT0G	8	0	0	0.245
CONT3G	8	0	3	0.275
CONT7G	8	0	7	0.239
CONT14G	8	0	14	0.219
CONT21G	8	0	21	0.326
TREAT3G	8	2.5	3	0.153
TREAT7G	8	2.5	7	0.159
TREAT14G	8	2.5	14	0.172
TREAT21G	8	2.5	21	0.190

Cadmium concentration (Cd (mg L^−1^)), days of exposure (time (days)), and within-treatment group average genetic diversity (He) were also reported

**Fig. 4:**
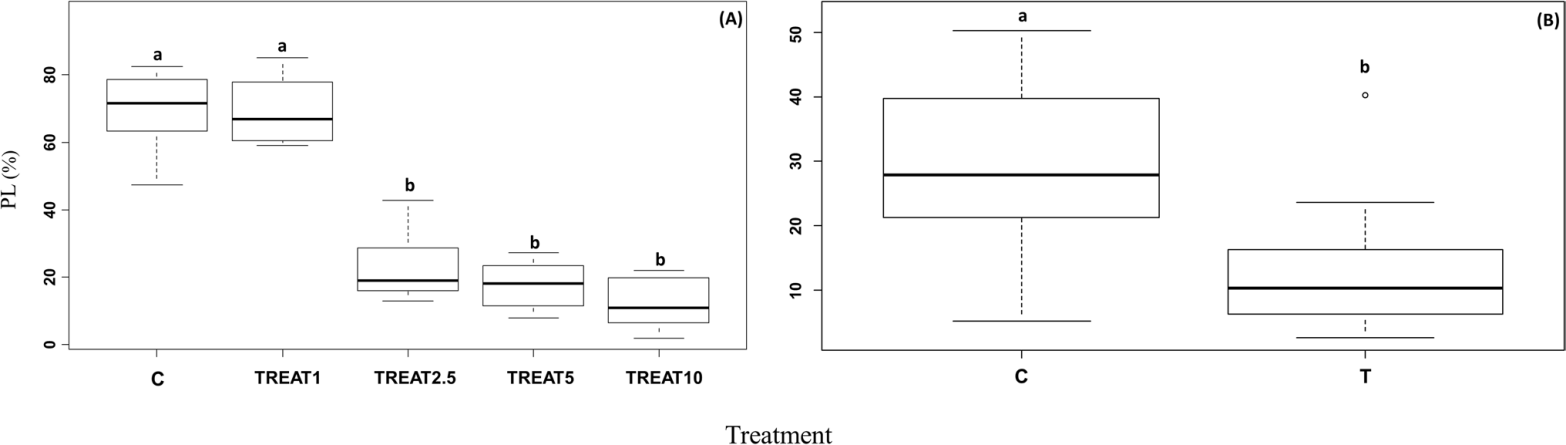
Boxplot showing the differences in the percentage of polymorphic loci composition PL (%) for experiment 1 (**A**) and experiment 2 (**B**). Treatments having the same letter are not significantly different from each other

The genomic template stability analysis showed a consistent reduction of the percentage of polymorphic loci for the group of treated samples excluding TREAT1 (Fig. 4A, *p* < 0.001). The number of SpL was higher for the group formed by controls and TREAT1 compared to the TREAT2.5, TREAT5, and TREAT10 (24 and 8, respectively). The genetic diversity within each sampling group varied from 0.326 for CONT21G and 0.153 for T03G in experiment 2 (Table 2). The analysis of genomic template stability confirmed the results of experiment 1, showing a reduction of the percentage of polymorphic loci for the treated group (Fig. 4B, *p* < 0.001). The number of SpL was 17 for the control group, whereas no specific loci were shown for the treated ones.

As for the AMOVA, the greatest percentage of the total genetic variation was due to among sampling group differences (52.38%, *p* < 0.001). Among all hypothetical cluster of sampling groups, the one formed by control and the treated with 1 mg L^−1^ of Cd (CONT-TREAT1), and the group of the plant exposed to 2.5 mg L^−1^, 5 mg L^−1^, and 10 mg L^−1^ (TREAT2.5- TREAT5- TREAT10), accounted for the highest percentage of among-groups variation 63.38%; *P* < 0.0001; Table 3). The higher level of total genetic variation in experiment 2 was for within the sampling group (70.8%, *p* < 0.022; Table 3). Among the hypothetical grouping of sampling groups, control plants vs treated plants accounted for the high percentage of variation among groups (see Table 3).

**Table 3:** Analysis of molecular variance

Source of variation	d.f	Sq	Vc	% of variation
**Experiment 1**				
Among grouping of sampling groups	1	780.45	29.95	63.38
Among sampling groups within groupings	3	59.87	0.29	0.61
Within sampling groups	47	799.63	17.01	36.00
Total	51	1639.94	47.25	
**Experiment 2**				
Among grouping of sampling groups	1	197.74	3.76	12.19
Among sampling groups within groupings	7	447.13	5.25	17.00
Within sampling groups	63	1377.88	21.87	70.81
Total	71	2022.74	30.89	

The table shows degrees of freedom (d.f.), sum of squared deviations (Sq), variance component estimates (Vc), and percentages of total variance contributed by each component (% of variation)

## Discussion

Our results provided useful and novel information for the direct quantification of the genotoxic effect of Cd-polluted waters by using AFLP fingerprinting on *M. aquaticum*.

Root and shoot growth was reduced in experiment 1 at 1 mg L^−1^ Cd (8,89 µM), in agreement with results from other studies showing that the first symptoms of toxicity were typically in solutions containing Cd at low micromolar concentrations (Andresen et al. 2013; Kovacik et al. 2017). Shoots and roots showed the same susceptibility to the presence of Cd, as demonstrated by the similar values of their EC_50ext_. Despite this, the two organs did not show the same level of Cd accumulation, thus suggesting different sensibilities to the internal metal concentrations. Since Cd accumulation was higher in roots than in shoots, this latter organ proved to be more susceptible to Cd toxicity (as quantified by its significantly lower EC_50int_ compared to the one of the roots). This outcome cannot be ignored in optimizing the tests; therefore, shoots were chosen for the genotoxic analysis to get more reliable results on the effect of Cd. For the designing of eco-toxicological tests, initial screenings need to be undertaken to choose the most suitable organ. Generally, roots of land plants are used for practical reasons (Aksoy, 2017), thus raising doubts not only about their validity as the most sensitive part but also on the reliability of the exposure methods. Obviously, plants exposed to Cd-contaminated waters are mostly the macrophytes, with the whole body in contact with the pollutant. This feature cannot be overlooked and makes the effects of the metal on such organisms hardly comparable with those on land plants.

Comparing the biometrical data to the molecular information, the first treatment concentration at which both the effect on growth and on DNA variation were simultaneously significant was as high as 2.5 mg L^−1^. This result indicated that metal-induced depression of plant growth did not necessarily imply a more dangerous genotoxic effect in the low Cd concentration zone. Precisely, the DNA-fingerprinting approach revealed a consistent separation of sampling groups in terms of the partition of molecular variance at treatments with 2.5 mg L^−1^, 5 mg L^−1^, and 10 mg L^−1^ of Cd. The plausible effect of Cd in varying the AFLP profiles among samples was also corroborated by the identification of 24 SpL loci for the group formed by control plants and plants treated at lower concentration (1 mg L^−1^). On the other hand, eight SpL loci were characteristic for the samples treated at 2.5 mg L^−1^, 5 mg L^−1^, and 10 mg L^−1^. Cadmium treatment seemed to affect the levels of genetic diversity of the treatment groups also since lower average genetic diversity values were shown for those sampling groups treated with the highest Cd concentrations (e.g., 5 mg L^−1^ and 10 mg L^−1^; Table 2). Moreover, the reduction of polymorphisms was detected for the treated group, by it having a different profile in AFLP fingerprinting when compared to the control plants. As revealed from other studies, the variation in genetic profiles, the appearance or disappearance of new specific loci, and the reduction of the genomic template stability may be the consequence of (i) genomic rearrangements in the primers binding sites or (ii) structural changes in the DNA sequences, induced by the genotoxic effect of different organic or inorganic substances (Liu et al. 2005; Rocco et al. 2014).

The effect of the Cd concentration of 2.5 mg/L on growth, accumulation, and AFLP profiles was evaluated in a time-dependent experiment (experiment 2). Concerning plant elongation and metal concentration in roots and shoots, experiment 2 confirmed the same results observed in the concentration-dependent one, as shown in Figs. 1 and 3. The two organs showed a similar Cd-induced decrease in growth (around 70% reduction of length increment at the end of the experiment for both the organs); nonetheless, shoots displayed a lower metal accumulation than roots and, accordingly, a higher sensitivity to the presence of the metal inside its tissues. Therefore, shoots were confirmed to represent a better candidate than roots for eco-toxicological tests. In both organs, a significant Cd-induced decrease in growth was observed after seven days. However, plant Cd accumulation occurred after three days, but apparently at a harmless concentration for growth. Looking at the molecular data, again, the results obtained from AFLP profiles did not match with plant growth measurements, this time with an unexpected vice versa compared to experiment 1. Cadmium treatment induced variation in the DNA-fingerprinting profiles, evidencing a genotoxic effect, already after three days but without a significant growth depression. Therefore, for shorter times and higher doses in respect to the critical threshold of experiment 1, the harmful effect of the metal on the plant genome was already present, thus corroborating the unreliability of eco-toxicological tests based only on plant growth already supposed by the results of experiment 1. Particularly, the AMOVA analysis showed that a large portion of the molecular variance is significantly explained by keeping the control samples separated from the treated ones. Also, the identification of 17 loci specific for the control plants confirmed that the genotoxic effect of 2.5 mg L^−1^ Cd treatment may become evident from the first days of the experiment. As in previous studies, DNA-fingerprinting profiles generated from plants exposed to increasing concentrations of phytotoxic inorganic substances revealed appearing and disappearing bands in comparison to control samples (Liu et al. 2009). Moreover, in our experiment, AFLP profiles variation induced by time-dependent Cd exposure was reflected by changes in DNA profiles and by reduction of the genome template stability after only three days, indicating a high sensitivity of the molecular approach in the identification of genotoxic effects of medium–low concentration of Cd in water. Our results corroborate other earlier studies (Labra et al. 2003; Tanee et al. 2018), reporting the high sensitivity of molecular approach than classic genotoxic trials since they are capable of detecting DNA changes that may not manifest themselves as biometrical mutations.

## Conclusions

*Myriophyllum aquaticum* was shown to be a suitable model system for the investigation of Cd genotoxicity through the AFLP fingerprinting profile. Our joint experiments in concentration and in time allowed us to identify two of the combinations of dose (2.5 mg L^−1^) and exposure (3 days) for Cd-polluted waters beyond which concern for environmental genotoxic danger could arise. Moreover, our results showed that a metal-induced reduction of plant growth does not necessarily imply DNA damage and, more worryingly, vice versa. Consequently, there is an urgent need to revise those more classic eco-toxicological tests based only on plant growth. Such tests, despite being easy to use and cost-effective, can underestimate the risk associated with not detected Cd-induced mutations in the genome of living organisms that can have concerning implications in the long term.

## Data Availability

The datasets used and/or analyzed during the current study are available from the corresponding author on reasonable request.

## References

[CR1] Aina R, Labra M, Fumagalli P, Vannini C, Marsoni M, Cucchi U, Bracale M, Sgorbati S, Citterio S (2007). Thiol-peptide level and proteomic changes in response to cadmium toxicity in *Oryza sativa* L roots. Env Exp Bot.

[CR2] Aksoy Ö (2017) Detection of environmental mutagens through plant bioassays. *Plant Ecology-Traditional Approaches to Recent Trends. InTech Open*, 9–23. 10.5772/intechopen.69274

[CR3] Ali H, Khan E, Sajad MA (2013). Phytoremediation of heavy metals – concepts and applications. Chemosphere.

[CR4] Andresen E, Mattusch J, Wellenreuther G, Thomas G, Arroyo Abad U, Küpper H (2013). Different strategies of cadmium detoxification in the submerged macrophyte *Ceratophyllum demersum* L. Metallomics.

[CR5] Apel K, Hirt H (2004). Reactive oxygen species: metabolism, oxidative stress, and signal transduction. Annu Rev Plant Biol.

[CR6] ATSDR U (2007) CERCLA priority list of hazardous substances. http://www.atsdrcdcgov/cercla/07listhtml

[CR7] ATSDR U (2015) Toxicologial profiles, toxic substances portal. http://www.atsdrcdcgov/toxprofiles/indexasp

[CR8] Barzanti R, Colzi I, Arnetoli M, Gallo A, Pignattelli S, Gabbrielli R, Gonnelli C (2011). Cadmium phytoextraction potential of different *Alyssum* species. J Hazard Mater.

[CR9] Bettarini I, Colzi I, Coppi A, Falsini S, Echevarria G, Pazzagli L, Selvi F, Gonnelli C (2019). Unravelling soil and plant metal relationships in Albanian nickel hyperaccumulators in the genus *Odontarrhena* (syn *Alyssum* sect *Odontarrhena*, Brassicaceae). Plant Soil.

[CR10] Beyersmann D, Hartwig A (2008). Carcinogenic metal compounds: recent insight into molecular and cellular mechanisms. Arch toxicol.

[CR11] Brack W, Dulio V, Agerstrand M, Allan I, Altenburger R, Brinkmann M (2017). Towards the review of the European Union Water Framework Directive: recommendations for more efficient assessment and management of chemical contamination in European surface water resources. Sci Total Environ.

[CR12] Ceschin S, Bellini A, Scalici M (2020) Aquatic plants and ecotoxicological assessment in freshwater ecosystems: a review. Environ Sci & Pollut Res 1–14 https://doi.org/10.1007/s11356–020–11496–3 10.1007/s11356-020-11496-3 PMC7838074 33244691

[CR13] Colzi I, Doumett S, Del Bubba N, Fornaini J, Arnetoli M, Gabbrielli R, Gonnelli C (2011). On the role of the cell wall in the phenomenon of copper tolerance in *Silene paradoxa* L. Env Exp Bot.

[CR14] Colzi I, Rocchi S, Rangoni M, Del Bubba M, Gonnelli C (2014). Specificity of metal tolerance and use of excluder metallophytes for the phytostabilization of metal polluted soils: the case of *Silene paradoxa* L. Environ Sci & Pollut Res.

[CR15] Colzi I, Lastrucci L, Rangoni M, Coppi A, Gonnelli C (2018). Using *Myriophyllum aquaticum* (Vell) Verdc to remove heavy metals from contaminated water: better dead or alive?. J Environ Manag.

[CR16] Coppi A, Lastrucci L, Cappelletti D, Cerri M, Ferranti F, Ferri V, Foggi B, Gigante D, Venanzoni R, Viciani D, Selvaggi R, Reale L (2018). AFLP approach reveals variability in *Phragmites australis*: implications for its die-back and evidence for genotoxic effects. Front Plant Sci.

[CR17] Doyle JJ, Doyle JL (1990). Isolation of plant DNA from fresh tissue. Focus.

[CR18] Excoffier L, Smouse PE, Quattro JM (1992). Analysis of molecular variance inferred from metric distances among DNA haplotypes: application to human mitochondrial DNA restriction data. Genetics.

[CR19] Galardi F, Corrales I, Mengoni A, Pucci S, Barletti L, Barzanti R, Arnetoli M, Gabbrielli R, Gonnelli C (2007). Intra-specific differences in nickel tolerance and accumulation in the Ni-hyperaccumulator *Alyssum bertolonii*. Environ Exp Bot.

[CR20] Hassan SH, Van Ginkel SW, Hussein MA, Abskharon R, Oh SE (2016). Toxicity assessment using different bioassays and microbial biosensors. Environ Int.

[CR21] Hossain Z, Huq F (2002). Studies on the interaction between Cd^2+^ ions and nucleobases and nucleotides. J Inorg Biochem.

[CR22] IARC (International Agency for Research on Cancer) (2016) IARC monographs on the evaluation of carcinogenic risks to humans. Volumes 1–115. https://www.monographsiarcwhoint/list-of-classifications/

[CR23] Jin YH, Clark AB, Slebos RJ, Al-Refai H, Taylor JA, Kunkel TA, Resnick MA, Gordenin DA (2003). Cadmium is a mutagen that acts by inhibiting mismatch repair. Nat Genet.

[CR24] Khan MA, Ghouri AM (2011). Environmental pollution: its effects on life and its remedies. Journal of Arts, Science & Commerce.

[CR25] Kleinjans JC, van Schooten FJ (2002). Ecogenotoxicology: the evolving field. Environ Toxicol Pharmacol.

[CR26] Kováčik J, Babula P, Hedbavny J (2017). Comparison of vascular and non-vascular aquatic plant as indicators of cadmium toxicity. Chemosphere.

[CR27] Labra M, Di Fabio T, Grassi F, Regondi SMG, Bracale M, Vannini C, Agradi E (2003). AFLP analysis as biomarker of exposure to organic and inorganic genotoxic substances in plants. Chemosphere.

[CR28] Lastrucci L, Lazzaro L, Dell’Olmo L, Foggi B, Cianferoni F (2018). Impacts of *Myriophyllum aquaticum* invasion in a Mediterranean wetland on plant and macro-arthropod communities. Plant Biosyst.

[CR29] Liu W, Li PJ, Qi XM, Zhou QX, Zheng L, Sun TH, Yang YS (2005). DNA changes in barley (*Hordeum vulgare*) seedlings induced by cadmium pollution using RAPD analysis. Chemosphere.

[CR30] Liu W, Yang YS, Li PJ, Zhou QX, Xie LA, Han YP (2009). Risk assessment of cadmium-contaminated soil on plant DNA damage using RAPD and physiological indices. J Hazard Mater.

[CR31] Meudt HM, Clarke AC (2007). Almost forgotten or latest practice? AFLP applications, analyses and advances. Trends Plant Sci.

[CR32] Monteiro MS, Lopes T, Mann RM, Paiva C, Soares AMVM, Santos C (2009). Microsatellite instability in *Lactuca sativa* chronically exposed to cadmium. Mutat Res Genet Toxicol Environ Mutagen.

[CR33] Nei M (1987). Molecular evolutionary genetics.

[CR34] OECD (2014). OECD TG 238 Guidelines for the testing of chemicals sediment-free. Myriophyllum spicatum toxicity test.

[CR35] Ohe T, Watanabe T, Wakabayashi K (2004). Mutagens in surface waters: a review. Mutat Res.

[CR36] Orchard AE (1981). A revision of South American Myriophyllum aquaticum (haloragaceae), and its repercussions on some Australian and North American species. Brunonia.

[CR37] Panda BB, Panda KK (2002) Genotoxicity and mutagenicity of metals in plants. Physiology and Biochemistry of Metal Toxicity and Tolerance in Plants, pp 395–414. https://doi.org/10.1007/978–94–017–2660–3_15

[CR38] Qi XM, Li PJ, Wan LIU, Xie LJ (2006). Multiple biomarkers response in maize (*Zea mays* L) during exposure to copper. J Environ Sci.

[CR39] Rocco L, Valentino IV, Scapigliati G, Stingo V (2014). RAPD-PCR analysis for molecular characterization and genotoxic studies of a new marine fish cell line derived from *Dicentrarchus labrax*. Cytotechnology.

[CR40] Saitou N, Nei M (1987). The neighbor-joining method: a new method for reconstructing phylogenetic trees. Mol Biol Evol.

[CR41] Salameh E, Shteiwi M, Al Raggad M, Singh PV (2018). Waste water treatment. Water Resources of Jordan.

[CR42] Schneider S, Roessli D, Excoffier L (2000) Arlequin: a software for population genetics data analysis Version 2000. http://www.anthrounigech/arlequin PMC2658868 19325852

[CR43] Schwarzenbach RP, Escher BI, Fenner K, Hofstetter TB, Johnson CA, Von Gunten U, Wehrli B (2006). The challenge of micropollutants in aquatic systems. Science.

[CR44] Slatkin M (1995). A measure of population subdivision based on microsatellite allele frequencies. Genetics.

[CR45] Surgun-Acar Y, İşkil R, Ceylan K, Ceylan Y (2018) Genotoxicity assessment of heavy metals (Zn, Cr, Pb) on strawberry plants using rapd assay. Fresenius Envir Bulletin 27(4):2483–2491. http://www.hdlhandlenet/11772/1665

[CR46] Tamura K, Filipski A, Kumar S (2013). MEGA6: molecular evolutionary genetics analysis version 60. Mol Biol Evol.

[CR47] Tanee T, Chaveerach A, Sudmoon R, Teanma J, Ragsasilp A, Sirikhansaeng P (2018). Heavy metal accumulation and DNA changes in plants around an electronic waste dumpsite suggested environmental management plan. Environ Claims J.

[CR48] US Environmental Protection Agency (US EPA) (2015) Regulated drinking water contaminants. https://wwwepagov/sdwa/drinking-water-contaminant-human-health-effects-information

[CR49] Valverde M, Trejo C, Rojas E (2001). Is the capacity of lead acetate and cadmium chloride to induce genotoxic damage due to direct DNA-metal interaction?. Mutagenesis.

[CR50] Wang L, Gao Y, Wang X, Qin Z, Liu B, Zhang X, Wang G (2021). Warming enhances the cadmium toxicity on macrophytes Myriophyllum aquaticum (Vell.) Verd. seedlings. Environ Poll.

[CR51] Zhu Y, Costa M (2020). Metals and molecular carcinogenesis. Carcinogenesis.

